# Report on the Medical College

**Published:** 1842-09

**Authors:** 


### THE WESTERN JOURNAL.

Vol. VI.—No. III.

LOUISVILLE, SEPTEMBER 1, 1842

### REPORT ON THE MEDICAL COLLEGE.

A document bearing the above title recently appeared in one of the newspapers of this city, as part of the proceedings of the city council, and has, we understand, been distributed in the form of extras through the city and abroad over the country.

We have perused the so-called Report with all the care that such a document demanded, and with an interest not a little heightened by the circumstances under which it appeared. Its object was to procure the transfer of the Medical Institute, its grounds, apparatus, &c., from the present Board of Managers to the "Trustees of the Louisville College." Yet strange to say, the Committee from whom it emanates, almost forgetting the Managers, proceed to heap charges and opprobrious epithets on the present faculty of the Institute! However cogent these were to the Committee, and however pertinent to their design, they were deemed totally unworthy of consideration by a majority of the Council, who peremptorily rejected the report. The whole paper is conceived in the worst possible spirit, which, notwithstanding the attempted disguise, is so plain that he who runs may read. It is wholly *ex parte* in its character, and full of gross misrepresentations and misstatements. It is not our purpose, however, to enter into an examination of this document now. The Faculty of the Institute or the Board of Managers (we are not informed which), have in preparation a reply which, it is understood, will be both full and explicit. When it appears we shall endeavor to lay such portions of it before our readers as will enable them to see the matter in its true light.

The individuals at the bottom of this movement, and the motives by which they are actuated, are alike understood and appreciated. It proceeds from certain factious and disappointed members of the profession here, who from out their holes and hiding-places have kept up a series of petty annoyances for years. But in this instance they evince a degree of *malignity* and *moral turpitude* that is rarely witnessed among men professing to be honorable. In procuring the publication of this report, *they carefully concealed the fact of its having been rejected by the City Council,* nor was such statement made until called for through the public prints. Conduct like this will meet with the hearty execration of every honest man, and with that retributive justice which, however tardy, is ever sure.

The friends of the Institute (which is the true object of hatred) have in the meanwhile nothing to fear. The blow thus intended to inflict a fatal wound will recoil on the heads of its enemies. The Faculty were never more united and harmonious; they are firmly seated in the confidence of the community, a confidence founded on their worth as private individuals, and their ability as public teachers, and are filled with the same vigor and the same enthusiasm, which in the short space of five years have carried the school from inconsiderable beginnings and made it, in point of numbers and influence, the third in the Union. The citizens of Louisville look with pride and affection on the Institute, the noble monument of their own munificence, and will mete out prompt punishment to those who attempt to check it in its swift career to glory and renown, or blight the brilliant hopes that cluster so thickly around it. C.

